# EquiMoves: A Wireless Networked Inertial Measurement System for Objective Examination of Horse Gait

**DOI:** 10.3390/s18030850

**Published:** 2018-03-13

**Authors:** Stephan Bosch, Filipe Serra Bragança, Mihai Marin-Perianu, Raluca Marin-Perianu, Berend Jan van der Zwaag, John Voskamp, Willem Back, René van Weeren, Paul Havinga

**Affiliations:** 1 Inertia Technology B.V., 7521 AG Enschede, The Netherlands; mihai@inertia-technology.com (M.M.-P.); raluca@inertia-technology.com (R.M.-P.); berendjan@inertia-technology.com (B.J.v.d.Z.); 2 Department of Equine Sciences, Faculty of Veterinary Medicine, Utrecht University, 3584 CM Utrecht, The Netherlands; F.M.SerraBraganca@uu.nl (F.S.B.); W.Back@uu.nl (W.B.); r.vanweeren@uu.nl (R.v.W.); 3 Rosmark Consultancy, 6733 AA Wekerom, The Netherlands; info@rosmark.nl; 4 Department of Surgery and Anaesthesia of Domestic Animals, Faculty of Veterinary Medicine, Ghent University, 9820 Merelbeke, Belgium; 5 Department of Computer Science, Pervasive Systems Group, University of Twente, 7522 NB Enschede, The Netherlands; p.j.m.havinga@utwente.nl

**Keywords:** horse, gait analysis, lameness, IMU, optical motion capture, agreement analysis

## Abstract

In this paper, we describe and validate the EquiMoves system, which aims to support equine veterinarians in assessing lameness and gait performance in horses. The system works by capturing horse motion from up to eight synchronized wireless inertial measurement units. It can be used in various equine gait modes, and analyzes both upper-body and limb movements. The validation against an optical motion capture system is based on a Bland–Altman analysis that illustrates the agreement between the two systems. The sagittal kinematic results (protraction, retraction, and sagittal range of motion) show limits of agreement of ±2.3 degrees and an absolute bias of 0.3 degrees in the worst case. The coronal kinematic results (adduction, abduction, and coronal range of motion) show limits of agreement of −8.8 and 8.1 degrees, and an absolute bias of 0.4 degrees in the worst case. The worse coronal kinematic results are most likely caused by the optical system setup (depth perception difficulty and suboptimal marker placement). The upper-body symmetry results show no significant bias in the agreement between the two systems; in most cases, the agreement is within ±5 mm. On a trial-level basis, the limits of agreement for withers and sacrum are within ±2 mm, meaning that the system can properly quantify motion asymmetry. Overall, the bias for all symmetry-related results is less than 1 mm, which is important for reproducibility and further comparison to other systems.

## 1. Introduction

Lameness in horses can be defined as an alteration of the normal gait due to a functional or structural disorder of the loco-motor system, and can commonly be attributed to orthopedic pain [1]. It is by far the most expensive health issue in the equine field, with economic costs estimated between $680 million and $1 billion in the USA alone [2]. It has been demonstrated that equine veterinarians spend up to 40% of their working time assessing lameness [3]. Currently, most veterinarians rely on subjective visual examination of gait to detect movement asymmetries that are the common clinical sign of lameness. However, subjective lameness assessment has been shown to have some substantial drawbacks, most due to the limitations of human visual symmetry perception [4] and the bias effect [5], which ultimately leads to a poor agreement between veterinarians [6,7,8,9].

Objective quantification of lameness has been a topic of investigation in the field of equine research for several years now [10]. The solutions that were devised can be broadly divided into two categories: kinetic methods and kinematic methods. Kinetic methods analyze the forces resulting from movement, whereas kinematic methods analyze movement of internal and external body segments during locomotion.

Force platforms were among the first instruments used for objective lameness assessment [11], and are still described today as the “gold standard” for kinetic gait analysis. Force platforms measure the ground reaction force (GRF), as exerted on a limb during the stance phase, in three dimensions [12]. Force platforms are very precise and accurate instruments, but the data collection process is laborious and time-consuming: since only very few strides are captured as the horse moves over the limited surface of the force platform, several runs are necessary to collect the necessary number of strides. To address this problem, force-measuring horse shoes [13,14] and a force-measuring treadmill [15] were developed. However, these are not yet widely available as a practical tool for daily gait analysis.

For kinematic analysis, optical motion capture (OMC) systems [16,17] have been applied successfully [18,19]. These systems use reflective markers attached to the body of the subject and several (infra-red) cameras distributed across the room that track the 3D position of the markers. These systems are highly accurate and precise: the average position error is usually better than a few millimeters [17,20]. Generic motion parameters that can reveal early signs of lameness have been defined and validated [19,21,22]. Therefore, OMC systems are considered the “gold standard” for kinematic analysis [23]. However, for a full-body capture of a horse moving through a large volume, a significant number of OMC cameras and supporting infrastructure is needed, which incurs significant cost. Due to the scale, size, and complexity of the system, it is difficult to relocate it to other venues [23,24]; e.g., each time it is relocated, a calibration procedure needs to be followed by an expert in order to ensure data quality [17]. Therefore, the application of OMC systems is mostly limited to a fixed laboratory environment [23], and in some exceptional cases, large clinics.

Inertial measurement units (IMUs) [25] have been considered as a promising cost-effective alternative to OMC systems [8,26,27,28,29,30,31,32,33]. By rigidly attaching an IMU to a body segment, the orientation and (less reliably) the displacement of that body segment can be determined, making a set of these devices applicable as a kinematic measurement system [34]. Recent technological progress in microelectromechanical (MEMS) inertial sensors, combined with the abrupt drop of sensor prices thanks to the smartphone market, made small, low-cost, low-power, yet accurate IMUs available. Moreover, the development of low-power wireless technologies has made it possible to explore a new paradigm: wireless networked IMUs, which avoids the need to draw cables between the sensor locations.

This is what the EquiMoves system described in the present study provides: a wireless IMU-based solution that operates as a network capturing relevant horse motion variables at a high sample rate, at eight bodily positions, in real time and accurately synchronized.

In this article, we compare the EquiMoves system to a well-established OMC system for a set of well-defined kinematic horse locomotion parameters that both systems can produce. Since the EquiMoves system is designed for everyday use by a veterinarian, we performed experiments that best match that scenario. This precludes the use of a treadmill, for example, since that may influence the horse’s gait [35,36].

Although we use the OMC system as a source of an accurate ground truth, we do not consider it the gold standard per se; we are interested mainly in the level of agreement between the two systems and what may cause the observed differences. Both IMU-based and OMC-based systems have limitations; e.g., IMU systems suffer from drift errors that accumulate in the underlying integration calculations [25], while OMC measurements are influenced by the number of cameras that have each marker in view and the distance between each camera and each marker [17,20]. We chose to focus on differences caused by errors in the displacement and orientation measurements of both systems. Therefore, timing-related differences are eliminated by choosing one of the two systems as the timing reference for both.

Our contributions are as follows:

- We present a system that provides real-time wireless data acquisition with the potential for real-time feedback to the user. The system can handle various equine gaits (e.g., walk and trot). It registers and analyzes movements for all individual limbs, rather than only upper-body movement, for example, which is what the previously described systems [21,37] do. Most existing systems only work for a horse trotting along a straight line, and involve only sensors to measure asymmetry on the vertical displacement of head and/or pelvis [34,37]. In contrast, the EquiMoves system can be used for walking and trotting horses, for arbitrary paths, and can also measure limb motion.
- We provide an in-depth analysis of the agreement of the measurements of our system with an OMC system, looking at both upper-body and limb-related parameters.
- We provide a detailed description of a data-based algorithm for time synchronization and the alignment of position and orientation between the IMU and OMC data sets.

In the following sections, we describe the EquiMoves system in detail. We start by positioning it with respect to related work; then, we present background information on horse gait analysis. Subsequently, the main system components and methods are described. Next, we discuss the practical trials and results obtained for validating the system. We conclude with a discussion of the results, current limitations, and points of improvement as future work.

## 2. Related Work

The most widely-used method for equine gait analysis is currently visual examination, which is prone to the subjectivity and limited accuracy of the human eye [4]. This has been suggested as one of the reasons why agreement between equine vets evaluating lameness in horses is often low [8]. There is also a susceptibility to bias during the orthopedic exam [5]. Particularly in cases of mild lameness, studies showed that vets agreed whether a limb was lame or not in only 62% of the cases with mild lameness (i.e., having a mean score of less than 1.5 on the AAEP lameness scale [6,38]). When given the task of deciding whether the horse was lame and choosing the limb, vets agreed in less than 52% of the cases [6].

As discussed previously in Section 1, optical motion capture (OMC) systems have been applied successfully for kinematic gait analysis [18,19]. Generic motion parameters that can be used to assess the soundness of the horse objectively have been defined and validated for data produced by such systems [21,22]. Some of the disadvantages of these OMC systems are that they are quite expensive and mostly confined to a laboratory [23,24,28].

Those limitations can pose problems for practitioners who wish to make these assessments in the field, which includes outdoor locations. Apart from practical considerations, it can also be very important in general to perform such experiments in the horse’s familiar environment [39]. Another disadvantage is that an OMC system is limited to a specific motion capture volume, constrained by space and equipment [23]. Thus, unless that volume is large, only a small number of strides can be recorded for gait analysis [23,28,40]. Additionally, OMC systems use reflective markers that need to stay in view of a sufficient number of the OMC system’s cameras: it is not always possible to prevent occlusions (particularly self-occlusions), which results in missing or less-reliable data [24,41].

An alternative to OMC systems is the use of inertial measurement units (IMUs) [42,43]. These devices can be attached at key locations on the body of the subject and measure acceleration and angular velocity using accelerometers and gyroscopes, respectively (both are inertial sensors). Using these measurements, the kinematic variables like displacement and orientation of the body at these locations can be determined [34].

IMUs have been applied extensively and successfully for human motion capture applications, particularly in healthcare [23,40,42,44,45] and sports [46,47,48,49,50,51]. In equine research, inertial sensor systems are also quickly gaining popularity [34,52,53], particularly for objective lameness assessment [8,26,27,28,29,30,31,32,33]. Some older systems only used a single-axis accelerometer and/or gyroscope, which means that those systems rely only on one-dimensional data. Therefore, for those systems, the rotation of the sensors during movement and variation in sensor attachment influence the signals and their quality [26]. More recent works use full IMUs on several locations of the body [26,31,54,55].

For human motion capture applications, IMU technology has been validated using OMC systems [23,49,50,56,57,58,59] and normal video recordings [60]. So far, the IMU-based horse gait analysis systems have only been sparsely evaluated: there is work comparing IMU-based systems with subjective assessment [7,8,61,62,63], there is work comparing IMU-based systems with force platform systems [62,64,65], and there are studies comparing between IMU-based kinematic systems [30,66,67]. However, the agreement of IMU-based and OMC-based kinematic horse gait analysis systems is barely explored: earlier work is either limited to stride timing or frequency [28], limited to upper-body [32,34], limited to limb measurements [68,69], or performs no agreement analysis [34,70,71]. In this work, we perform an extensive comparison of our IMU-based system with a well-established OMC system, for both limb and upper-body parameters.

There are three known initiatives in the market to provide portable sensor-based systems for assisting equine veterinarians with gait analysis, which are related to the EquiMoves system presented here. A well-known commercial system is the Lameness Locator from the company Equinosis (Columbia, MO, USA) [27,72]. The Lameness Locator is based on three sensors: two single-axis accelerometers on the head and sacrum (see Section 3.1), and one single-axis gyroscope on the right forelimb, for determining the stance or swing phase of the diagonal pair of limbs in trot. The measured vertical acceleration of the torso is used to determine asymmetries in head and pelvic position between left and right halves of stride in trot. Due to the limited number and type of sensors, the Lameness Locator is usable only in trot, and the produced results relate only to the upper body of the horse.

Another commercial system is GaitSmart Pegasus (Codicote, Hitchin, Hertfordshire, UK) [73], which mainly uses the gyroscope in two directions attached to the limbs, so it focuses on limb-related parameters. Some of the main outputs of Pegasus are the temporal phase-lag between limb cycles and the (angular) range of motion. However, none of these parameters are fully validated with respect to the gold-standard laboratory equipment [69], and their biological significance still needs to be investigated with respect to their correlation with lameness. For the Pegasus system, the angular range of motion was compared with an OMC system similar to ours in an earlier study by Roepstorff et al. [69]. Their results show a rather large bias between the two systems, yet the agreement between the two systems looks quite good. However, as we demonstrate in the discussion of our own results in Section 7.3, Roepstorff et al. analyzed the agreement on a trial level, rather than on a per stride basis, which means that their agreement results are somewhat diluted, potentially painting an unrealistic picture of the true error. Additionally, the agreement was calculated on a mixed data set of walk and trot trials, thereby diluting the effect of gait.

Finally, the EquiGait system [74] (Brickendon, Hertford, Hertfordshire, UK) uses IMUs on the upper body to measure either gait symmetry, back movement, or horse–rider interaction (with one IMU on the rider). Alternatively, a mobile phone can be used over the pelvic area. Although the EquiGait system is validated [34] for the upper-body parameters that it can produce, it is currently not used to assess limb-related parameters (to the best of our knowledge).

## 3. Background

In this section, we provide the necessary background on equine locomotion theory.

### 3.1. Horse Anatomy

**Figure 1:**
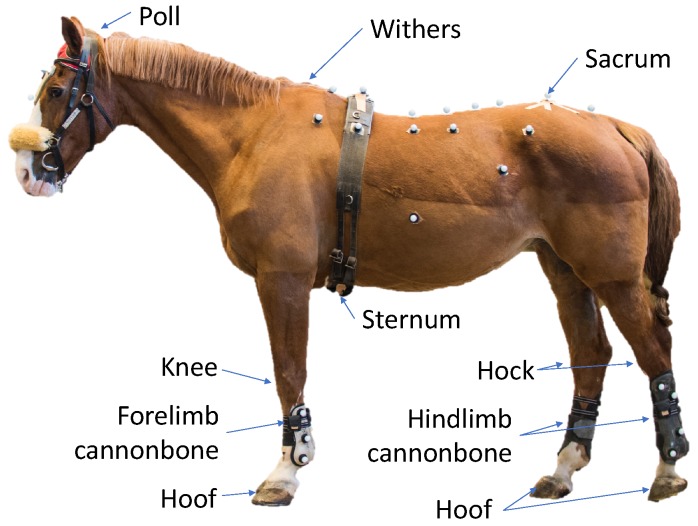
Relevant anatomical positions on a horse.

Figure 1 shows a horse equipped with the EquiMoves hardware. Parts of the anatomical structure of the horse that are relevant to this work are annotated.

The *poll* position is on top of the head of the horse between the ears. The *withers* is a position on the horse’s back between the shoulder blades. The *sternum* is the breastbone in the middle of the horse’s chest, which is located on the underside of the horse, just behind the forelimbs. The *sacrum* or *pelvis* is on the back of the horse above the hindlimbs. The *cannon bone* is the bone just beneath the knee in the forelimbs and beneath the hock in the hindlimbs.

**Figure 2:**
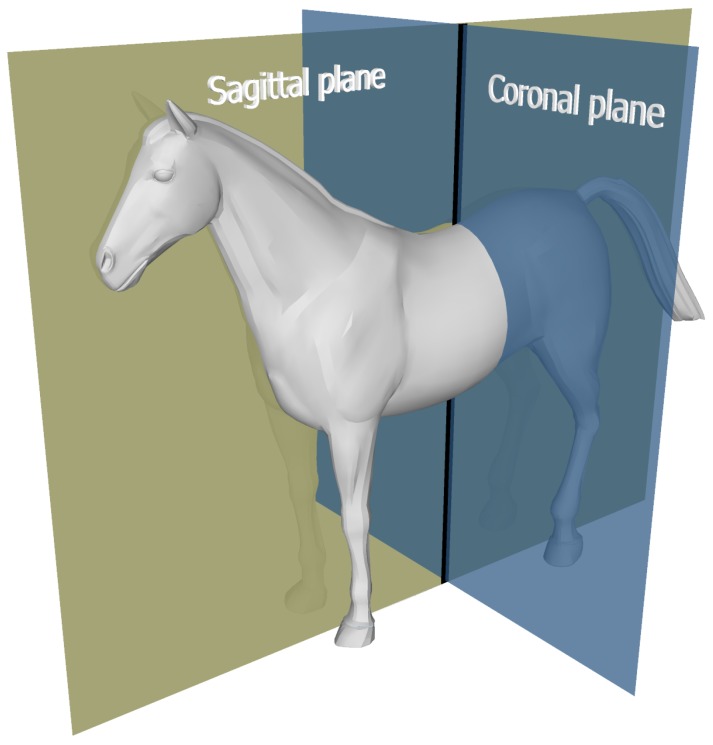
Planes for horse motion.

As shown in Figure 2, horse motion can be analyzed in the following imaginary planes (among others):

- sagittal (anterior–posterior) plane: divides body in left/right; describes forward backward motion
- coronal (medial–lateral) plane: divides body in front/back; describes left/right sideways motion

### 3.2. Horse Locomotion Parameters

#### 3.2.1. Stride Timing

For a single limb, strides are delimited by the moment the hoof touches the ground; the *stride duration* is the time that passes between these events. The *stride frequency* is the reciprocal of the stride duration. Strides can be divided in a *swing phase* and a *stance phase*. The swing phase starts at the moment the hoof is lifted from the ground (the *hoof-off* moment) and ends when it touches the ground once more (the *hoof-on* moment). The stance phase is the period of time the hoof is on the floor.

#### 3.2.2. Limb Angle Parameters

The *protraction* is the forward extension of a limb, while the *retraction* of a limb is the backward extension [75]. The protraction and retraction angles of the cannon bone are defined here as the sagittal (forward/backward) swing angles of that bone during a stride relative to vertical. The (sagittal) range of motion (ROM) is the full angular distance between retraction and protraction. This definition matches what is used by Roepstorff et al. [69]. The limb can also have a coronal angle during a stride; when the limb is tilted outwards, it is called an *abduction angle*, and, when it is tilted inwards, it is an *adduction angle*. In this article, the protraction and retraction angles are determined at the respective hoof-on and hoof-off moments. In contrast, the abduction and adduction angles are determined as the coronal angle extrema.

**Figure 3:**
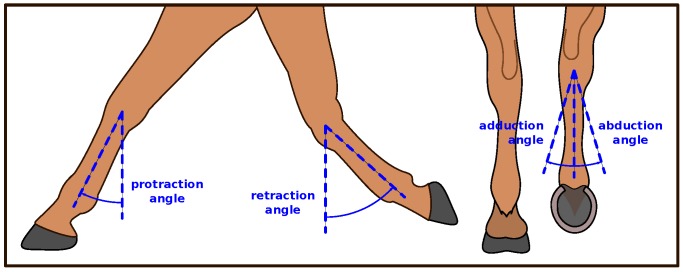
Forelimb protraction and retraction angles, and hindlimb adduction and abduction angles.

The protraction and retraction angles for the forelimbs are shown schematically in Figure 3 on the left side. The sagittal angles for the hindlimbs are defined identically. On the right, Figure 3 also shows the coronal abduction/adduction angle for one hindlimb.

#### 3.2.3. Upper-Body Symmetry Parameters

**Figure 4:**
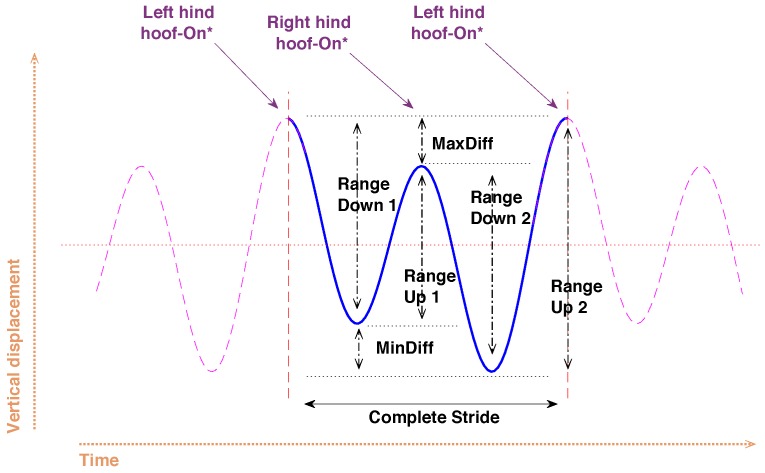
Upper-body vertical displacement annotated with symmetry parameters. The timing of *hoof-on* instances is approximate.

Upper-body symmetry parameters can be calculated at several upper-body locations: poll, withers, sternum, and sacrum. At each of these positions, the vertical displacement of the body is measured. At each stride, the upper body typically moves up and down two times for the successive left and right limb steps, so there are two peaks and two troughs in the signal, as shown in Figure 4. If the horse moves perfectly symmetrically, these extrema are at the same level [21].

The following parameters are derived from the vertical displacement signal:

- Max_diff: the difference between the two peaks (${\mathrm{Max}}_{1}$ and ${\mathrm{Max}}_{2}$) of the vertical displacement signal [21].
- Min_diff: the difference between the two troughs (${\mathrm{Min}}_{1}$ and ${\mathrm{Min}}_{2}$) of the vertical displacement signal [21].
- ${\mathrm{Range}}_{\mathrm{up},1/2}$: The upwards range or amplitude is the difference between a trough and the next peak in the vertical displacement signal. Since there are two troughs, there are two separate values.
- ${\mathrm{Range}}_{\mathrm{down},1/2}$: The downwards range or amplitude is the difference between a peak and the next trough in the vertical displacement signal. Since there are two peaks, there are two separate values.
- $\mathrm{Range}\_{\mathrm{diff}}_{\mathrm{up}}$: the difference between the two upwards ranges.
- $\mathrm{Range}\_{\mathrm{diff}}_{\mathrm{down}}$: the difference between the two downwards ranges.

The upwards or downwards *symmetry index* (SI) can be derived from these parameters as follows [21]:

(1) $${\mathrm{SI}}_{\mathrm{up}/\mathrm{down}}=\frac{{\mathrm{Range}}_{\mathrm{up}/\mathrm{down},1}-{\mathrm{Range}}_{\mathrm{up}/\mathrm{down},2}}{\max\{{\mathrm{Range}}_{\mathrm{up}/\mathrm{down},1},{\mathrm{Range}}_{\mathrm{up}/\mathrm{down},2}\}}.$$

A value of ±1 indicates maximum asymmetry (the sign depending on the affected limb) and a value of 0 indicates perfect symmetry.

## 4. System Overview

**Figure 5:**
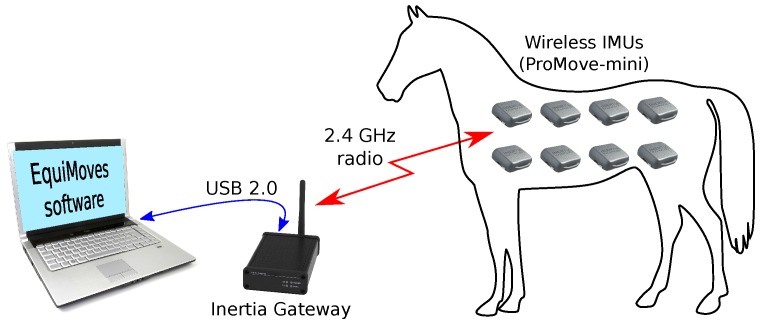
System overview. IMU: inertial measurement unit.

In this section, we provide an overview of the EquiMoves system in terms of hardware and software. Figure 5 provides a schematic overview of the system.

### 4.1. IMU Hardware

**Figure 6:**
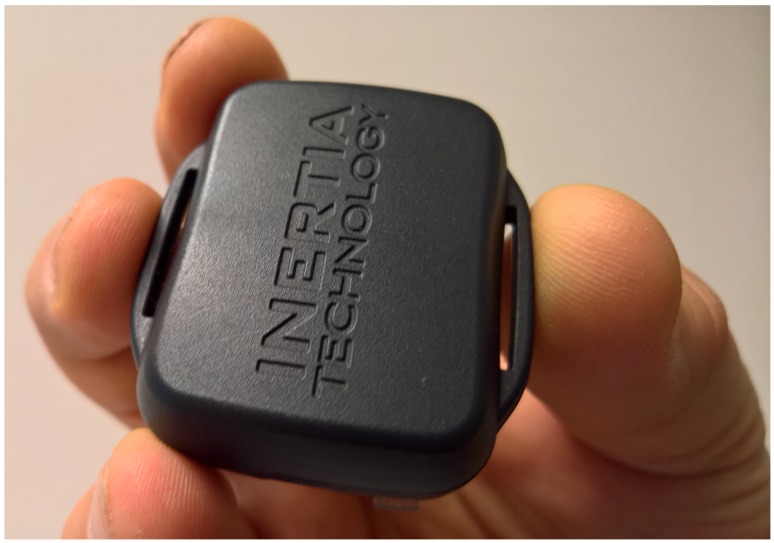
ProMove-mini.

As shown in Figure 5, the hardware of the EquiMoves system consists of up to eight ProMove-mini wireless IMUs (Inertia Technology B.V., Enschede, The Netherlands) [76] for capturing motion data, one wireless receiver (the Inertia Gateway), and a (laptop) computer or tablet that runs the EquiMoves software. A ProMove-mini IMU (see Figure 6) weighs 20 g and features a set of 3D digital sensors providing acceleration, angular velocity (gyroscope), and magnetic field intensity (compass). The ProMove-mini has two separate aligned accelerometers that can simultaneously measure low-*g* acceleration with a range of ±16 *g* and high-*g* acceleration with a range of ±400 *g*. With this two-sensor setup, a single fused signal can be obtained with a high precision and range, even when the registered acceleration exceeds ±16 *g*—for example, at foot impacts during trot or canter. The gyroscope can measure angular velocity within range of $\pm 2000{\;}^{\circ }/s$. More limited ranges can be configured for all sensors to attain higher precision.

The wireless network operates in the 2.4 GHz ISM band using a proprietary protocol. The Inertia Gateway acts as the coordinator in a network of ProMove-mini nodes with a star topology; all nodes in the network communicate only directly with the gateway. The transmission range is up to 30 m in ideal conditions. Sensor data from all IMUs is streamed through the gateway to the computer running the EquiMoves software. The IMUs are continuously and actively time-synchronized within a precision of 100 ns, practically guaranteeing sampling at precisely the same time instance in all measurement points.

The gateway can handle a wireless network of up to 39 ProMove-mini IMUs sampling and streaming data from all inertial sensors (accelerometers and gyroscope) at 200 Hz and the compass at its maximum sample rate of 100 Hz. When using fewer nodes, sampling rates up to 1 kHz are possible for the inertial sensors; i.e., the EquiMoves system could be configured for 1 kHz with the full network of eight nodes, which could be necessary for measuring gallop, for example. For trot, 200 Hz is ample [70,77].

To mitigate packet loss in the wireless network and to facilitate performing measurements (briefly) outside the wireless transmission range, the sensor data can be stored simultaneously on the on-board 2 Gb SD-card memory of each node. After the experiment, the recorded data can be retrieved wirelessly through the gateway or through USB by connecting each IMU to the computer.

Figure 1 shows a horse equipped with the EquiMoves hardware. Refer to Section 5.3 for details on horse instrumentation.

### 4.2. Motion Processing

**Figure 7:**
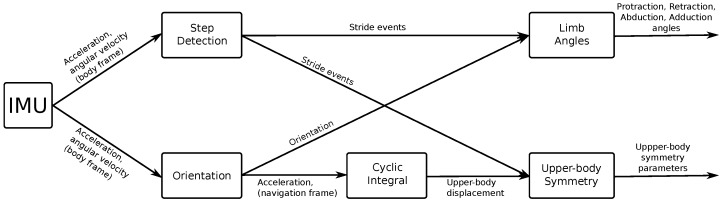
The EquiMoves motion processing framework.

The EquiMoves motion processing software (see Figure 5) receives the raw sensor data from the IMU network through USB and computes the relevant parameters described in Section 3.2 for analyzing the horse’s gait with respect to lameness and performance assessment. This is a complex process that involves the following main processing steps, as depicted in Figure 7. This section provides a high-level overview of motion data processing for the EquiMoves system; the details are described in Section 6.

During **Step Detection**, the raw IMU sensor data from each limb is used to identify the exact motion cycles broken down into strides, and each stride into its stance and swing phases. The main challenges here are to detect the *hoof-on* and *hoof-off* moments (see Section 3.2.1) with maximum accuracy, from the inherently noisy sensor signals. A detailed analysis of the developed detection algorithms, and the comparison with existing methods, is provided in an earlier study by Bragança et al. [78]. The outputs of this step are: *hoof-off* and *hoof-on* moments, and the *stride duration*, distinguishing between *stance* and *swing* durations.

In the **Orientation** processing step, the raw IMU sensor data is fused to obtain 3D orientation information. The angular velocity from the gyroscope sensor is integrated into a quaternion orientation. Measurements from the accelerometer are used as a means of correcting the orientation with respect to the direction of gravity, thus limiting the gyroscope drift. Although a compass sensor is available (see Section 4.1), it is not used in this framework: practical experience with the compass sensor demonstrates that it is often too unreliable, especially when used inside a concrete or steel building. This means that there is no absolute heading reference. However, such a heading reference is not strictly necessary, since the absolute heading of the horse is currently not relevant to the calculated gait parameter. With the obtained 3D orientation, the acceleration measured by the IMUs is rotated from the body reference frame of each IMU to a navigation reference frame in which the *z*-axis is aligned with gravity. The outputs of this step are the IMU orientation represented as a quaternion, and the IMU acceleration represented in the navigation frame.

In the **Limb Angles** processing step, the orientation of the IMU sensor on the limbs is used to determine the angle of the limb at that position relative to the vertical. The orientation at the start and end of the swing phase is used to determine the axis of the sagittal (forward) rotation. Using a so-called swing–twist decomposition (refer to Section 6.7), both the sagittal and coronal (sideways) swing angles of the limb are calculated for the whole swing phase. The outputs of this phase are: *protraction and retraction angles*, *abduction and adduction angles* (see Section 3.2.2).

In the **Cyclic Integral** processing step, upper-body velocity and displacement are computed in a manner similar to the **Limb Displacement** step, but instead of zero-velocity reset per step, a cyclic integration process is applied, as described in [34]. Through this process, the unbounded integration drift is removed by subtracting the mean of the current and adjacent strides at each integration step.

In the **Upper-Body Symmetry** processing step, the upper-body displacement is segmented per stride, and the position and magnitude of the signal extrema are determined and used in the calculation of the symmetry parameters (see Section 3.2.3). The outputs of this processing step are, for each location (withers, sacrum, sternum, and poll), as follows: Min_diff, Max_diff, $\mathrm{Range}\_{\mathrm{diff}}_{\mathrm{up}/\mathrm{down}}$, ${\mathrm{SI}}_{\mathrm{up}/\mathrm{down}}$.

## 5. Experiments

We analyze the relative performance of the IMU-based EquiMoves system by comparing its measurements to an OMC system. This section describes how the experiments were conducted.

### 5.1. Venue

**Figure 8:**
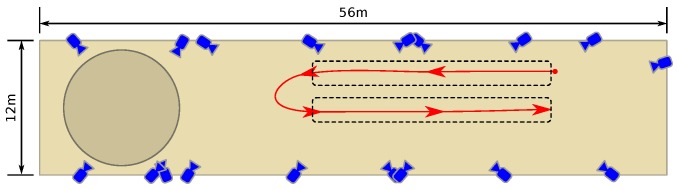
The experiment venue at Utrecht University including the approximate path of the horses.

Experiments were performed in the Equine Clinic of Utrecht University, at the Department of Equine Sciences. The clinic is equipped with an OMC system (Qualisys Oqus 700+, Qualisys AB, Göteborg, Sweden) with 18 infra-red camera’s (12 MP, 4096×3072 pixels) mounted at a height of approximately 8 m (aimed downwards) that record the position of reflective markers attached to the horse at key positions. Figure 8 shows a schematic overview of the room, including the location and aim of the cameras. The cameras are distributed across the hall by the manufacturer such that a subject is sufficiently in view of at least two cameras at all times. The relative precision of the OMC system after calibration as deployed was determined to be 1.9 mm.

### 5.2. Horses

As shown in Table 1, seven Warmblood clinic-owned mares with body mass between 512 and 593 kg, height at the withers in the range of 1.61 to 1.69 m, and age between 10 to 21 years were used for this study. The listed horse numbers are used in the results in Section 7. None of the subjects had a recent history of lameness.

### 5.3. Data Collection

**Figure 9:**
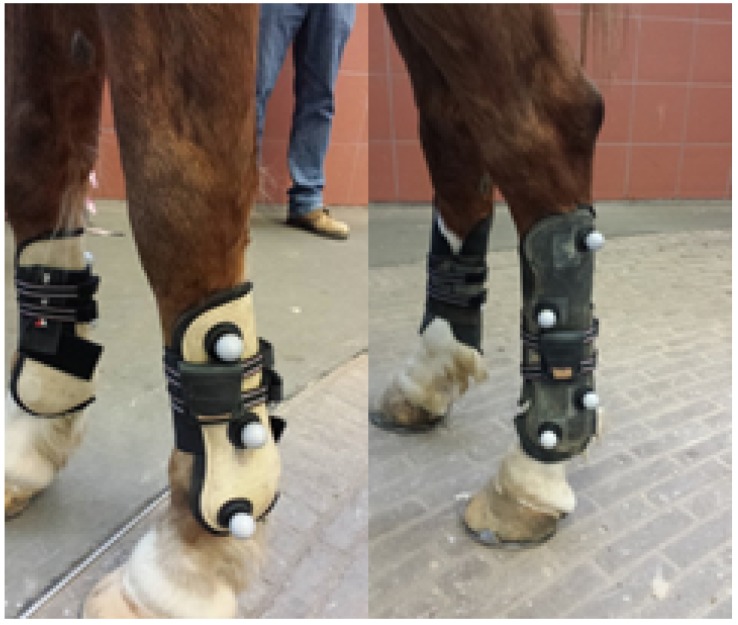
Placement of the IMU sensors and optical motion capture markers on fore- and hindlimbs.

**Figure 10:**
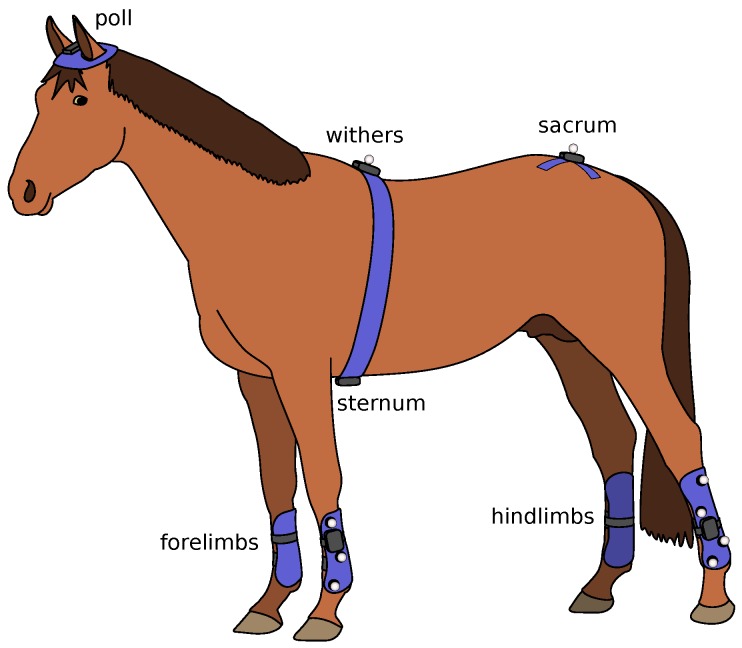
Overview of the equipment attached to each horse.

All subjects were instrumented with eight IMU sensors (refer to Section 4.1) on the poll, withers, sacrum, sternum, and each limb (refer to Section 3.1). On the limbs, each sensor node was firmly attached to the lateral aspect of each cannon bone using a custom-made holster (see Figure 9). At the withers and sternum of the horse, the sensor nodes were mounted on a girth, a cap was used at the poll, and double-sided tape was used at the sacrum. Figure 10 shows an overview of the sensor locations.

The sensors were configured with a sampling rate of 200 Hz. For the sensors attached to the limbs, the range for the low-*g* accelerometer was configured at its maximum range of ±16 *g* and the high-*g* accelerometer at a mid-level range of ±200 *g*, which is sufficient for trot. The sensors at the upper-body locations were configured with the low-*g* accelerometer at ±8 *g* for better accuracy.

During the experiment, the IMU sensors transmitted their samples wirelessly to a nearby laptop computer in real time. To prevent data loss, the data was also stored in the internal memory of each individual sensor (see Section 4.1). At the end of the experiment, any samples missing in the laptop’s data file were filled in from the internal memory of each sensor by downloading those wirelessly.

Several reflective markers were placed on the IMU sensor holsters on the limbs, so that the OMC system could determine both position and orientation at these locations (refer to Section 6.2). Four reflective markers (25 mm ⊘, spherical soft passive markers) were glued around each of the hindlimb-mounted IMUs, and three on the forelimbs also around the IMUs. Furthermore, three reflective markers were glued on the poll (head), three on the girth, and three on the pelvis (tuber sacrale, left and right tuber coxale). The IMU sensors on the upper body had only a single reflective marker located on or near the IMU sensor, which limited those OMC measurements to displacement only.

Motion capture data was recorded at 200 Hz using 18 infrared cameras (refer to Section 5.1) previously calibrated per manufacturer instructions to a global coordinate system. All trials were also recorded on normal video using standard equipment for retrospective analysis of the collected data. These video recordings were hardware-synchronized to the OMC system.

During the measurements, the three-dimensional coordinates of each marker were automatically tracked by the motion capture software (QTM, version 2.11a, Qualisys AB, Göteborg, Sweden). After each measurement, visual inspection of the 3D-tracked data confirmed that all markers were properly tracked and data was suitable for analysis. Measurements with poor marker tracking or irregular gait patterns were discarded and repeated.

Due to occlusions caused by the horse’s ears and due to the camera positioning at our venue, no marker could be placed directly on the IMU sensor at the poll location; therefore, the nearest marker was too far away from that sensor for a reliable comparison. This could have been achieved by performing this experiment on a treadmill. Nevertheless, it was our goal to perform this comparison overground, for which situation the EquiMoves system is designed and aimed to collect data. This means that the poll location measurements were excluded from our analysis.

All subjects were fitted with the instruments and were led by an experienced handler in walk and trot in a straight line while motion capture and IMU data was collected. Two experiments were performed for each horse at walk and trot, back and forth along a straight path, as shown by the arrow track in Figure 8.

## 6. Data Processing

We analyzed the relative performance of the IMU-based EquiMoves system by comparing its measurements to an OMC system. We are mainly interested in the differences caused by the errors in the displacement and orientation measurements of both systems.

### 6.1. Missing Data

Our comparisons skip time instances where data from either system is missing. To prevent problems with time synchronization (see Section 6.5.1) between the two systems, any gaps in the data of either system were filled with placeholder samples. As explained in Section 4.1, the EquiMoves system prevents data loss by storing a copy of the data locally on the SD-card in each node, which can be downloaded after the experiment. This means that the data is always complete for the duration of the experiment. The OMC system is not wireless, so packet loss is not an issue there. However, the reflective markers can become occluded, which means that an insufficient number of OMC cameras has the marker in view and the position of that marker cannot be determined at that time. This happened during some of our experiments, meaning that the OMC data set has minor gaps. Since the gaps were minor, these were skipped in the processing, rather than interpolated.

### 6.2. Orientation

The orientation of the IMU sensors was determined using an attitude and heading reference system (AHRS) algorithm by Valenti et al. [79]. At its core, it integrates the rotational velocity from the gyroscope sensor into an orientation represented as a quaternion. Measurements from the accelerometer are used as a means to correct the orientation with respect to the direction of gravity. In terms of Euler angles, this means that gyroscope drift errors in the pitch and roll angles are compensated. However, without an additional reference (e.g., the heading vector provided by a compass sensor, refer to Section 4), the yaw angle is left uncompensated. The static accelerometer filter gain $\bar{\alpha }$ for the Valenti algorithm was set to 0.0001 for these experiments (refer to Equation (61) in [79]).

In these experiments, orientation data for the OMC system is only available for the limbs, since, at those locations, several reflective markers were placed that together formed a so-called *rigid body*. For such a rigid constellation of markers, the OMC system can calculate the orientation and position for each captured camera frame. At those limb positions, the IMU was placed in the middle of the markers so that its displacement and orientation would directly match the movements of the OMC system’s rigid body.

For both systems, the orientation is represented in unit quaternions [79,80]. In essence, a unit quaternion can describe a rotation from one orientation to another around an axis or vector $v=\left[\begin{array}{ccc} v_{x} & v_{y} & v_{z} \end{array}\right]$ by an angle θ. Such a rotation quaternion $q_{r}$ is composed as follows:

(2) $$q_{r}={\left[\begin{array}{cc} \cos\frac{\theta }{2} & v\sin\frac{\theta }{2} \end{array}\right]}^{T}.$$

### 6.3. Position and Displacement

The OMC system produces position measurements for individual markers or a *rigid body*, as described in the previous section. From an absolute position, displacement can be readily calculated. In contrast, IMU systems need to integrate the acceleration twice to obtain displacement estimates. First, the orientation measurement is used to rotate the acceleration measurements from the sensor reference frame to a navigation reference frame, which has the *z*-axis aligned with gravity. Subsequently, the acceleration signal is integrated twice to successively obtain velocity and displacement values. As described in Section 6.8, a special integration algorithm is used to limit the drift in the integrals that arises from IMU measurement errors [25].

### 6.4. Noise Filtering

The acceleration, velocity, and angular velocity signals calculated from the position and orientation measurements produced by the OMC system were noisy (mostly spikes) (see, for example, Figure 12), likely due to the occasional marker occlusions and inherent to its position estimation algorithms. The same signals from the IMU were less noisy in most cases, but these were not completely noise-free either. Thus, to prevent the noise from having an effect on our agreement analysis, the data for both systems was first low-pass filtered using a fourth-order Butterworth filter with a cut-off frequency of 30 Hz. This is well beyond the highest frequency component of interest at two times the stride frequency (around 3 to 4 Hz) [81,82]. To prevent group delay from having an effect on our synchronization, zero-phase filtering was used at all times. The filter was applied to displacement and angular signals identically for both systems where necessary; more details are provided in subsequent sections.

### 6.5. Alignment with Camera Data

Both the EquiMoves IMU-based system (see Section 4.1) and the OMC system yield data sets that are intrinsically synchronized across all of their measurement locations. However, to compare the data between the two systems, the data sets need to be aligned in time and space. This was performed in post-processing.

#### 6.5.1. Time-Synchronization

At the time of data collection, there was no means available to achieve hardware-based time synchronization between the IMU and OMC systems. Therefore, time-synchronization between the two systems was achieved by calculating the correlation coefficient on a signal that could be readily obtained from both systems. The peak in the resulting correlation curve yields the lag between the two data sets. The clock skew (frequency difference) between the two systems was assumed to be negligible for the length of the experiment. Time-synchronization is therefore a matter of applying the calculated lag to the time stamps of one of the two data sets. This method is similar to what Howard et al. [83] use to synchronize their IMU and force platform signals.

We chose to use the angular velocity of one limb as input signal for the correlation, since this is easily and reliably calculated from both systems. For the IMU, this is the raw output from the gyroscope. For the OMC system, this can be calculated from the rigid body quaternion orientation q signal by *quaternion differentiation*.

Equation (3) describes the computation of the orientation of a gyroscope sensor at time $t_{k}$ by numerical integration of the angular velocity signal from that sensor [79,80] using sample period $\Delta t=t_{k}-t_{k-1}$. The angular velocity is represented in the sensor *body reference frame*. The orientation of the sensor is represented as a quaternion $q_{\omega }={\left[\begin{array}{cccc} q_{\omega ,1} & q_{\omega ,2} & q_{\omega ,3} & q_{\omega ,4} \end{array}\right]}^{T}$, which describes a rotation from the *global reference frame* to the sensor body reference frame. The integration process estimates the required quaternion derivative ${\dot{q}}_{\omega }\left(t_{k}\right)$ from the angular velocity $\omega ={\left[\begin{array}{ccc} \omega_{x} & \omega_{y} & \omega_{z} \end{array}\right]}^{T}$ in Equation (3b). The ⊗ operator is the *quaternion multiplication*:

(3a) $$\begin{array}{cc} q_{\omega }\left(t_{k}\right) & =q_{\omega }\left(t_{k-1}\right)+{\dot{q}}_{\omega }\left(t_{k}\right)\Delta t, \end{array}$$

(3b) $$\begin{array}{cc} {\dot{q}}_{\omega }\left(t_{k}\right) & =\frac{1}{2}q_{\omega }\left(t_{k-1}\right)⊗\left[\begin{array}{c} 0\\ \omega (t_{k}) \end{array}\right]. \end{array}$$

To obtain the angular velocity signal ω(t) from the existing quaternion orientation signal q(t) from the OMC system, the numerical integration of Equation (3) can be reversed into a numerical derivative as follows:

(4a) $$\begin{array}{cc} \left[\begin{array}{c} 0\\ \omega (t_{k}) \end{array}\right] & =2{\dot{q}}^{*}\left(t_{k}\right)⊗q\left(t_{k-1}\right), \end{array}$$

(4b) $$\begin{array}{cc} \dot{q}\left(t_{k}\right) & =\frac{q\left(t_{k}\right)-q\left(t_{k-1}\right)}{\Delta t}. \end{array}$$

**Figure 11:**
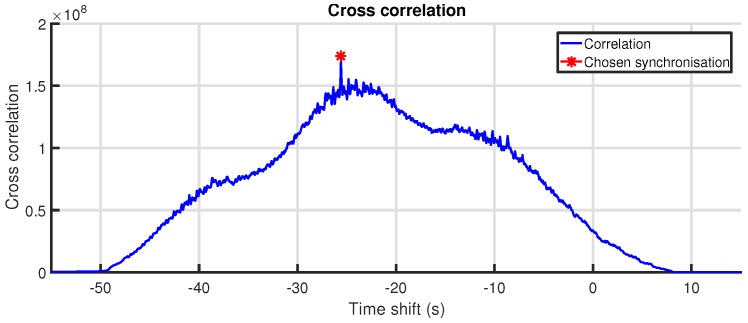
Example of the cross-correlation between angular velocity magnitude between IMU and optical motion capture (OMC).

**Figure 12:**
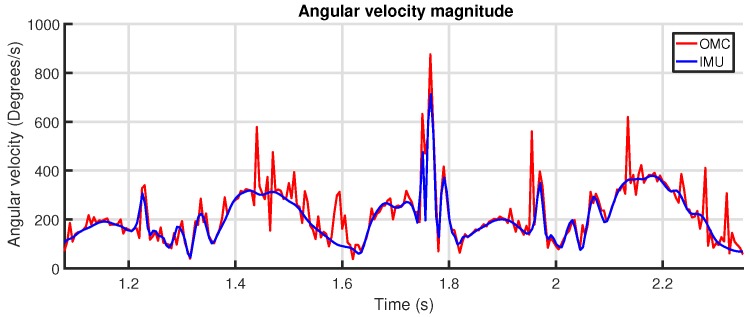
Example of the synchronization of angular velocity magnitude between IMU and OMC.

At this point, we have obtained an angular velocity signal ω(t) for each system. However, these signals are three-dimensional. To make these signals scalar and—more importantly—to remove the influence of the relative orientation between the IMU and OMC system data, the magnitude ∥ω(t)∥ (Equation (5)) is used as input for the correlation coefficient. An example of the correlation coefficient between two angular velocity magnitude signals is shown in Figure 11. Figure 12 shows the raw angular velocity magnitude signals after synchronization.

(5) $$∥\omega \left(t\right)∥=\sqrt{{\omega_{x}\left(t\right)}^{2}+{\omega_{y}\left(t\right)}^{2}+{\omega_{z}\left(t\right)}^{2}}$$

The time resolution of the peak location in the correlation coefficient is dictated by the sample frequency of both systems. In our case, these frequencies are identical at 200 Hz. To obtain a more accurate estimate of the lag between the two systems, the location of the peak is determined more precisely by quadratic interpolation over three points. By visual inspection, the accuracy of the achieved synchronization is estimated to be better than 1 ms. For the comparison between the two systems, the OMC signals were resampled to synchronize them with their IMU equivalents, based on the time lag found by the method described above.

#### 6.5.2. Orientation and Position Alignment

Before evaluating the high-level results, it is useful to compare the data sets at a lower level in terms of their position and orientation values. For this comparison, the data sets need to be aligned: the OMC system uses an absolute room-referenced coordinate system, whereas the IMU system uses a navigational reference frame that is not anchored to anything, apart from the *z*-axis, which is aligned with the gravity vector.

At the limb positions, the OMC system’s markers that form a rigid body and the IMU sensor were mounted rigidly on the same position (see Section 6.2), which means that the motion sequence measured by both systems will be identical, apart from a fixed relative translation and rotation between the two systems. Since the positions are deemed identical and only displacement values are compared, any translation between the two systems will be ignored. Therefore, we only need to find the rotation value, which should remain constant for the whole experiment, assuming that the physical attachment of the hardware does not change during the experiment. The data sets are assumed to be already synchronized in time by the procedure described in the previous section, so we can correctly pair measurements between the two systems.

To determine the rotation between the two systems, we need to have a signal from each system that depends on the orientation of its measurement reference frame. We use the angular velocity signals that we used for time synchronization in the previous section. In this case, we use the actual 3D signals and not their magnitude. The gyroscope measures the angular velocity in the sensor’s body reference frame, whereas the OMC angular velocity is calculated relative to the reference frame of the defined rigid body. Therefore, the rotation that maps the IMU’s angular velocity measurements to the corresponding signal calculated for the OMC system is equal to the rotation between the two systems, which is the desired rotation value.

We determined the translation and rotation between the two signals using the Kabsch algorithm [84]. This is a method for obtaining the optimal rotation and translation between two spatial structures defined by a set of paired points, which minimizes the distance between the paired points. To use this algorithm, we considered our angular velocity signals to be structures in 3D space. The individual samples are the points that define each structure. The points are paired per time instance between the systems.

**Figure 13:**
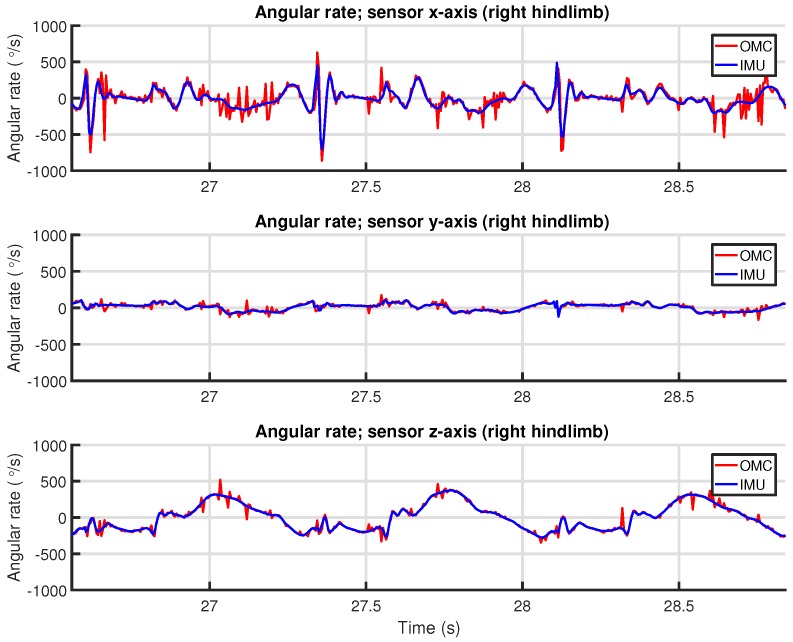
Example of the alignment of angular velocity between IMU and OMC.

The rotation resulting from the Kabsch algorithm directly describes the relation between the orientation of the IMU sensor reference frame and the orientation of the rigid body defined for that location by the OMC system. Note that, considering the nature and origin of the angular velocity signals and the fact that both signals describe the same motion sequence, there should be very little translation apart from some gyroscope bias. Applying this mapping to one of the angular velocity signals allows these to be compared directly, as shown in Figure 13.

As stated earlier in Section 6.4, OMC system signals can be very noisy, especially derivatives from such signals (as seen in Figure 13). Therefore, to prevent the alignment from being affected, we excluded periods where the absolute difference in the variance of the magnitude of the two signals exceeded a threshold of $1.0\times 10^{-3}\;{{(}^{\circ }/s)}^{2}$ over a window of 10 samples.

### 6.6. Hoof Event Detection

As described earlier in Section 1, this work focuses on the comparison of displacement and angular measurements. Using different hoof event timing for the two systems would distort the results and make it difficult to pinpoint what is causing the difference. Since we have pre-existing experience with extracting timing information from the IMU data, we used the IMU as the timing reference; e.g., the stride segmentation was performed based solely on the IMU data. We used the algorithm published earlier by Bragança et al. [78], denoted as Algorithm 1 therein.

### 6.7. Limb Angles

The orientation of the limbs at the cannon bone was determined for both the IMU and OMC systems using the procedures explained earlier in Section 6.2. Equally for both systems, this orientation was used to determine the sagittal and coronal limb angles (see Section 3.2.2) during each stride.

Since the orientation is determined in a global/room (OMC) or navigation (IMU) reference frame, the heading of the horse is part of the orientation. Therefore, it is necessary to distinguish the forward swing (sagittal) direction for each stride individually. This is performed by determining the rotation axis between the orientation at the *hoof-off* and *hoof-on* moments (refer to Section 3.2.1). This axis is perpendicular to the sagittal plane. To obtain this axis, we calculate the quaternion that transforms between these orientations:

(6) $$q_{r}=q_{on}⊗q_{off}^{*}.$$

The sagittal part of rotation is subsequently determined using a *swing–twist decomposition* [85] around the *z*-axis in the respective system’s global/navigation reference frame. The swing–twist decomposition is used to decompose a rotation $q_{r}$ into a rotation $q_{t}$ around a specified axis v (the twist) and the remaining rotation $q_{s}$ around another axis (the swing), so that:

(7) $$q_{r}=q_{t}⊗q_{s}.$$

In our case, the resulting twist quaternion $q_{t}$ is the component that consists of a rotation around the *z*-axis, and the swing quaternion $q_{s}$ is the residual rotation around a different (horizontal) axis, which is the sagittal part of the rotation we are looking for. The sagittal axis is subsequently extracted from the swing quaternion $q_{s}$ as its vector part v (see Section 6.2). Then, we perform another swing–twist decomposition—this time on each sample of the limb orientation signal for each stride. This is used to determine the twist rotation around the sagittal axis for the duration of each stride. The sagittal angle signal is readily extracted from the resulting twist quaternion signal (see Section 6.2). Subsequently, the sagittal axis is rotated 90 degrees around the *z*-axis to obtain the coronal axis. Again using a swing–twist decomposition, the coronal angle signal is determined.

A problem that arises with these calculations is the definition of where the sagittal and coronal angles are zero. A logical choice would be that these angles are zero when the limb—or rather the cannon bone (refer to Section 3.1)—is vertical. This situation is not trivial to determine from either the OMC or the IMU data sets. Since finding an accurate algorithm for this is outside the scope of this work, we defined a position in the stride that we can determine reliably on both systems and that still approximates the vertical orientation quite well. We chose to use the 50% of stance (mid-stance) moment; i.e., exactly in the middle of the stance phase of the stride. As long as both systems use this time-based reference, our results will not be biased by this definition. As before (see Section 6.6), we used the IMU as reference for stride segmentation; therefore, the derived mid-stance moment is exactly the same time instance for the IMU and OMC systems.

To implement this zero reference, the limb orientation $q_{r,m}$ is calculated as a rotation relative to the limb orientation at the mid-stance $q_{m}$ (Equation (8)). The resulting quaternion $q_{r,m}$ is used instead of the limb orientation $q_{r}$ itself in the process described above:

(8) $$q_{r,m}\left(t\right)=q_{r}\left(t\right)⊗q_{r}^{*}\left(t_{midstance}\right).$$

**Figure 14:**
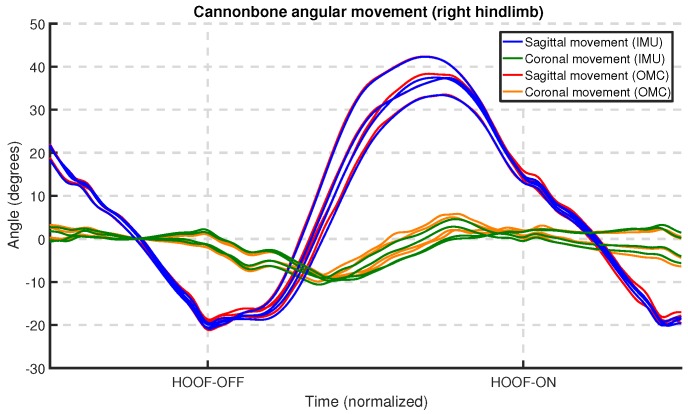
Example of the cannon bone angular motion for IMU and OMC systems.

To remove any noise, the angular signals were subsequently filtered using the filter described in Section 6.4. Finally, the protraction, retraction, adduction and abduction angles were determined as the coronal and sagittal angles at the respective hoof-on and hoof-off moments (see Section 3.2.2). Figure 14 shows an example of the limb angle signals resulting from this process; the plot shows several strides of the right hindlimb.

### 6.8. Upper-Body Symmetry

As described briefly in Section 3.2.3, the upper-body symmetry parameters are used to estimate to what extent the horse’s gait is symmetrical at key positions on the upper body of the horse. These parameters are entirely based on vertical motion. At each of these positions, the horse’s body moves up and down in a cyclic fashion exactly two times each stride. In sound (i.e., healthy) horses, this signal is symmetrical and the two maxima and minima are at the same level.

The vertical displacement of the relevant upper-body locations is readily calculated from the position measurements of the OMC system. In contrast, for the IMU-based EquiMoves system, the accelerometer data needs to be integrated twice to obtain a displacement value. As described earlier in Section 6.3, the accelerometer data was first rotated to a navigation reference frame, so that the *z*-axis acceleration lined up with the gravity vector. Subsequently, the vertical displacement was obtained using a cyclic integration method first described by Pfau et al. [34]. In essence, this subtracts the mean value of the current, present, and next stride from the acceleration and velocity, respectively, so that drift is effectively removed at each integration step. To remove any noise, the resulting displacement signal for both systems was low-pass-filtered using the filter described in Section 6.4.

The vertical displacement signal is not only determined by the horse locomotion itself. For both systems, this signal is also affected by an uneven or slanted floor surface. Additionally, the IMU signal is still subject to some drift, since the displacement resulting from the double cyclic integral is not mean-subtracted. To mitigate this problem, the displacement signal was high-pass-filtered using another fourth-order Butterworth filter. For maximum effectiveness and flexibility, this filter was tuned to the step-frequency of the horse, so that the cut-off frequency was optimized for the gait type. The stride frequency is determined as the reciprocal of twice the median time between two successive peaks in the displacement signal. The cut-off frequency for the filter was set to 2/3 of the stride frequency. The stride frequency was determined for all locations based on the vertical displacement at the sacrum, since it was determined most reliably there. For walk, the resulting cutoff frequency ranged from 0.5 Hz to 0.6 Hz over all experiments, and, for trot, it laid between 0.8 Hz and 0.9 Hz. To prevent group delay from having an effect on our synchronization, zero-phase filtering was used.

**Figure 15:**
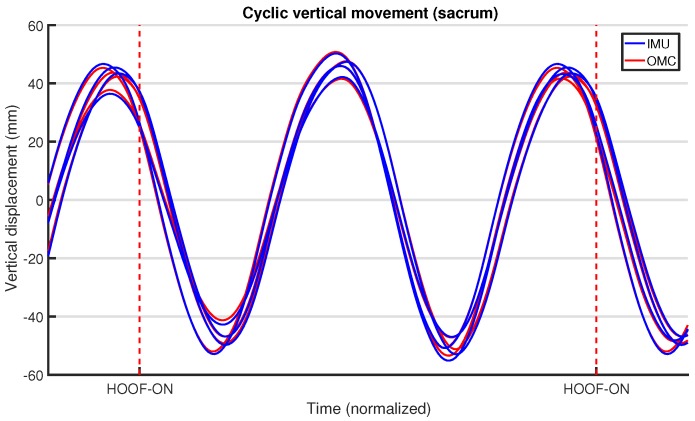
Example of the vertical displacement at the sacrum for IMU and OMC systems. The vertical dashed lines indicate the left hindlimb hoof-on moments used for stride segmentation.

The resulting vertical displacement signal was cut into stride-delimited segments. For each stride, the extrema were determined, and using these values the upper-body symmetry parameters were calculated (refer to Section 3.2). Figure 15 shows the vertical displacement at the sacrum for several strides for the IMU and OMC systems.

## 7. Results

### 7.1. Overview

We used Bland–Altman analysis (using the R package BlandAltmanLeh, version 0.3.1, RStudio, Boston, Massachusetts, USA) to study the agreement between measurements of the IMU and OMC systems [86,87]. This method allows for the comparison of two measurement systems that can potentially both have errors; no instrument is ever perfect. This method consists of making a scatter plot of the differences between the measurements of the two systems as a function of the mean of those measurements. The plot shows a line for the agreement bias as the mean difference between the systems, and two lines for the limits of agreement (LOAs) are plotted at a distance of 1.96 standard deviations above and below that bias line (see Section 7.3 and Section 7.4). Ideally, there is no bias (mean difference is zero) and the bias line is located at zero. The LOAs indicate the boundaries of the agreement interval in which 95% of differences are contained. This is a measure for the expected difference between the two systems based on the experiments. Ideally, the LOAs are very close to the bias line, indicating that the spread of the differences is very small in the majority of cases. Since there are multiple measurements (strides) per individual horse, confidence intervals were generated for the LOAs [88]. We included regression lines in the Bland–Altman plots to indicate whether the bias was (mostly) constant over the measurements in our data set and to visually assess homoscedasticity. Earlier work by Pfau et al. [67] presented the data in a similar way.

We also calculated the intra-class correlation (ICC) using the R package psychometric (version 2.2). ICC is a measure for the reproducibility/consistency of our measurements [87]. The ICC was calculated from a linear mixed model to account for repeated measurements, since we had the data calculated on a per stride basis. The horse ID was used as the grouping variable (random effect) and the ICC was calculated as $t_{00}/\left(t_{00}+\sigma^{2}/n_{j}\right)$, where $t_{00}$ is the variance of the intercept of the model, $\sigma^{2}$ is the residual variance for the model, and $n_{j}$ is the group size. The obtained values for the ICC will range between zero and one. A value of zero indicates a poor reproducibility between the two systems, and an ICC of one indicates a perfect reproducibility.

### 7.2. Low-Level Observations

To understand where differences between OMC and IMU measurements may originate, we first discuss observations on a few low-level signals that are produced as part of the signal processing performed to obtain these measurements (refer to Section 6).

**Figure 16:**
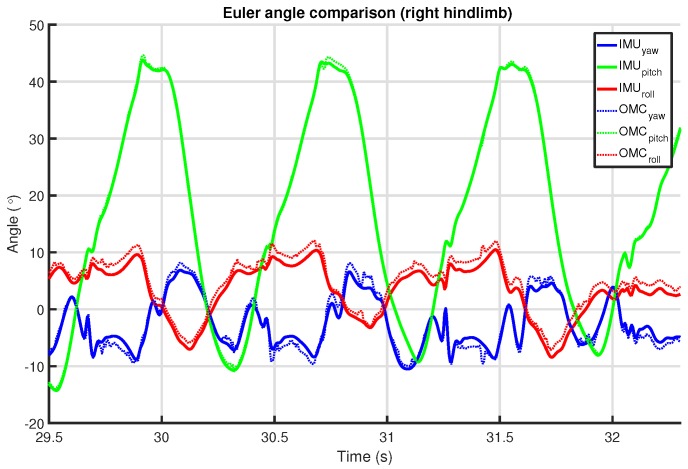
Example of the limb orientation match between IMU and OMC systems for the right hindlimb.

Figure 16 shows an example of the limb orientation measurements from the IMU and OMC systems represented in Euler angles. No additional filtering beyond what each system itself performs was applied. The orientations were aligned using the algorithm discussed in Section 6.5 in an effort to remove any static orientation difference. The example shows that the IMU orientation is more smooth/less noisy than the OMC orientation. The OMC orientation also shows some signal artifacts that are not present in the IMU output (e.g., at time 31.4 s).

We can only speculate as to what caused these differences. Since there are some small transients in the OMC output, it is plausible that at least some of these differences were caused by marker occlusions. On other occasions, high acceleration may influence the gravity vector tracking of the Valenti algorithm [79], which we used for the IMU (refer to Section 6.2). Another factor, which would mainly affect the coronal angle measurements for the limbs, is the depth perception of the OMC cameras [89]. This is likely aggravated by the manner in which these are installed in the clinic (i.e., high above the floor looking down). This is near-optimal for upper-body motion capture but is less suitable for measuring limb movements. Additionally, the three markers on the forelimbs were mounted almost colinearly (see Figure 9), which, as it turns out, is not optimal for reliably determining the rigid body orientation. In contrast, the hindlimbs have four markers in a rectangular shape.

Further evaluation of the OMC limb data shows that the results of the left forelimb were quite unreliable, particularly the rigid body orientation (refer to Section 6.2). The results suffer from full marker occlusions (i.e., missing position data for some of the markers), and as a consequence the rigid body orientation was noisy and at times implausible. The occlusions were likely caused by the handler obstructing the view of the cameras to the markers on that limb (i.e., the handler was too close to the horse). This is why the comparison for that location shows much poorer results than the other limbs. Therefore, the left forelimb was excluded from the summary results. However, we do present the specific results for this location to demonstrate the effect of this issue.

**Figure 17:**
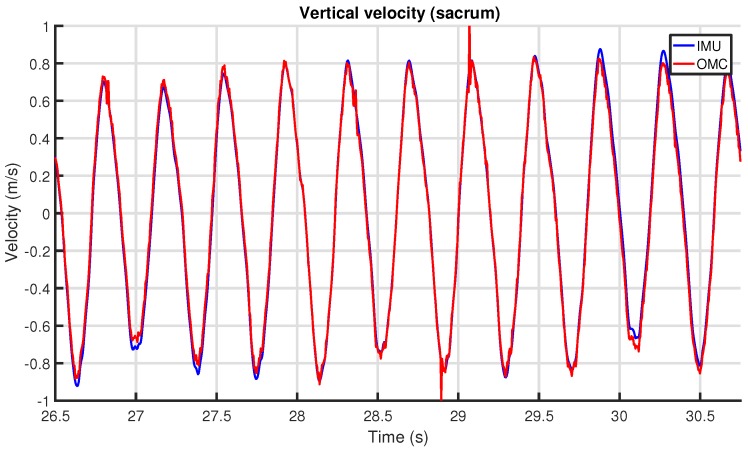
Example of the vertical velocity match between IMU and OMC systems for the sacrum.

Figure 17 shows an example of the match between the IMU and OMC measurements of the vertical velocity at the sacrum. A static offset in the IMU velocity is removed by subtracting the mean of the whole signal. The OMC signal directly results from differentiating the marker position. Both signals are otherwise unfiltered. Overall, the signals are very similar. However, while the signals line up almost perfectly in the middle, offset differences are visible at the beginning and end, likely due to drift in the IMU velocity. Additionally, there is noise in the OMC signal.

**Figure 18:**
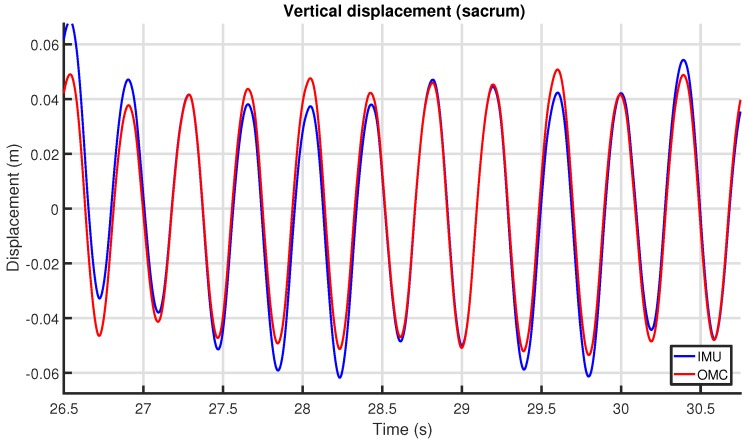
Example of the vertical displacement match between IMU and OMC systems for the sacrum.

The vertical displacement for the same experiment is shown in Figure 18. Noise in the OMC signal is no longer evident. IMU drift stands out much more, as is visible from the varying offset difference between the two signals. This drift was removed in the next step by the high-pass filter tuned to the stride frequency (see Section 6.8), which, after stride segmentation, resulted in the output of Figure 15.

### 7.3. Limb Angles

Table 2 presents a summary of the difference statistics for the limb angle signals used to calculate the limb angle parameters. When comparing the sagittal and coronal results in Table 2, the angle signals for the sagittal plane show less difference between the two systems when compared with the coronal plane. As discussed in Section 7.2, we speculate that the coronal angles for the OMC system were influenced by the depth perception of the OMC cameras and the manner in which these cameras are installed in the clinic.

**Figure A1:**
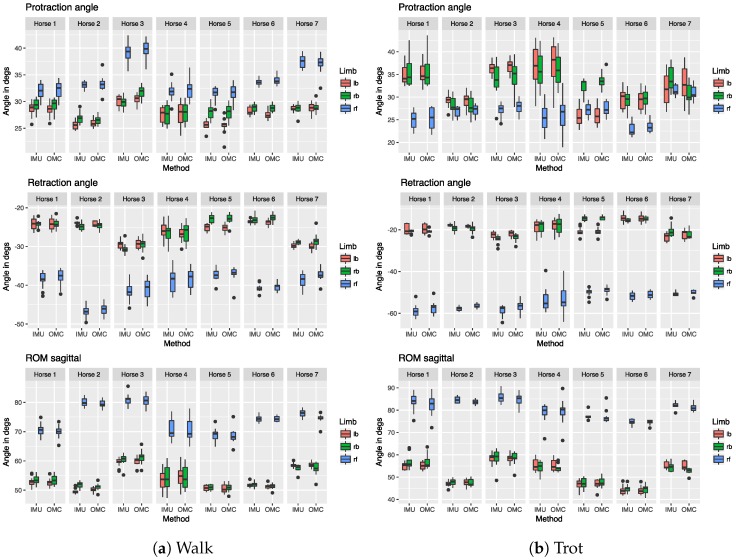
Box plots for the sagittal limb angles measured by IMU and OMC.

**Figure A2:**
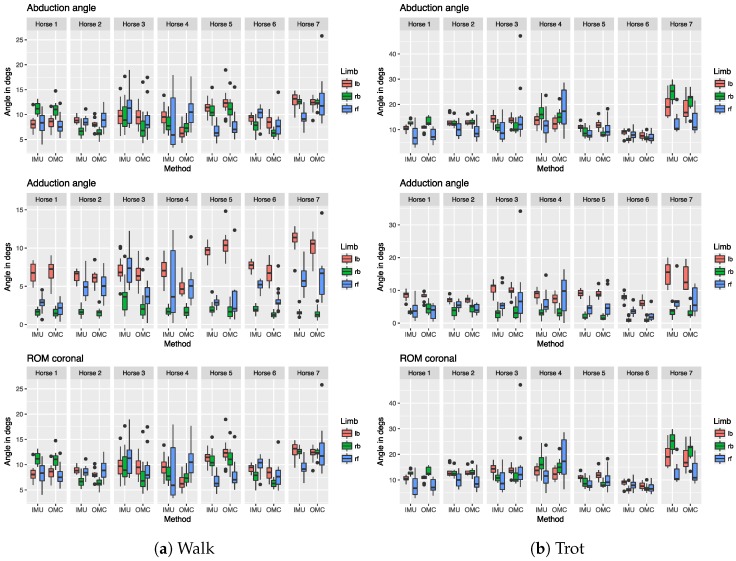
Box plots for the coronal limb angles measured by IMU and OMC.

Detailed box plots for the limb angle parameters clustered by gait and limb can be found in Appendix A in Figure A1 and Figure A2. The difference between fore- and hindlimbs for the angles in the sagittal plane is noteworthy: the higher range of motion in the sagittal plane has been described previously using IMU techniques [69]. We also observed a higher sagittal ROM at trot when compared to walk, also in line with previous publications [69].

While the per-horse variation is low (small height of the boxes in the box plots), which highlights the repeatability of our measurements, a large variation is noticeable between horses in all calculated limb parameters. Nevertheless, the analysis of variation in the measured parameters between horses is outside the scope of this manuscript: future work should investigate the variation in the calculated parameters between horses in more detail.

**Figure 19:**
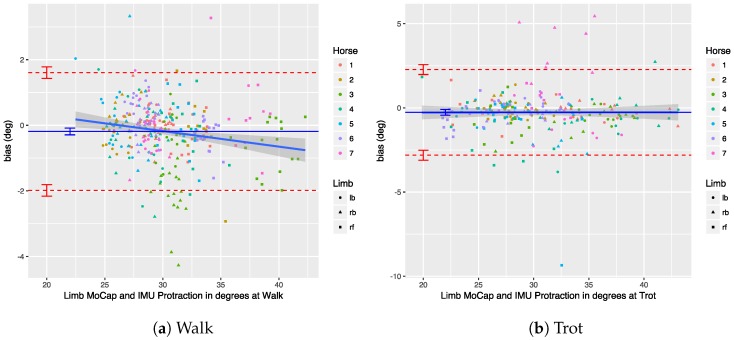
Bland–Altman analysis for protraction angles at walk and trot.

**Figure 20:**
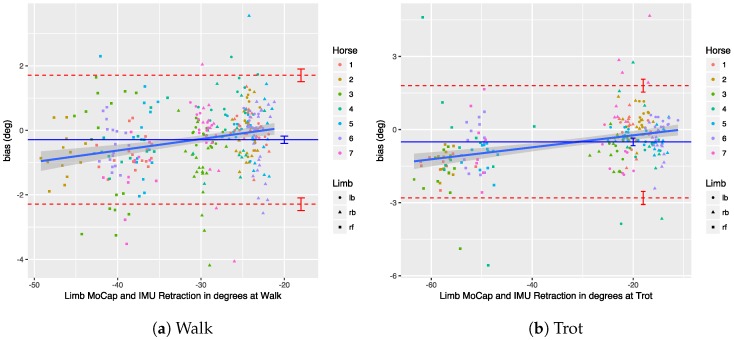
Bland–Altman analysis for retraction angles at walk and trot.

**Figure 21:**
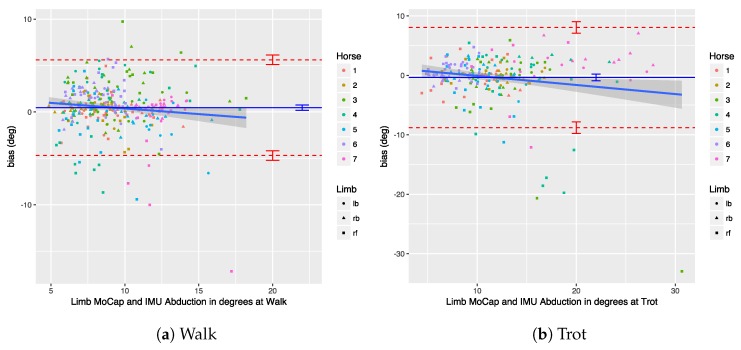
Bland–Altman analysis for abduction angles at walk and trot.

**Figure 22:**
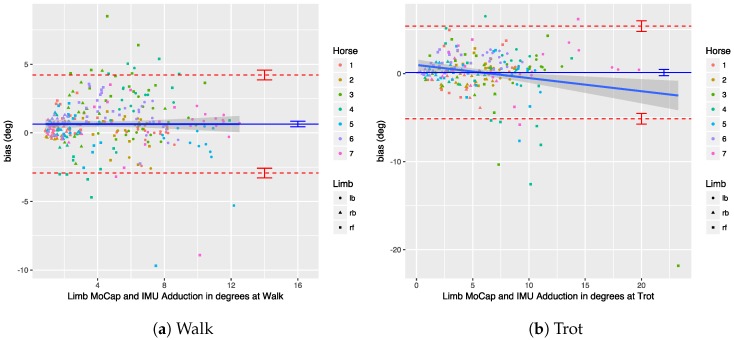
Bland–Altman analysis for adduction angles at walk and trot.

We performed a Bland–Altman analysis of the protraction, retraction, abduction, and adduction angles (see Section 3.2.2), respectively, in Figure 19, Figure 20, Figure 21 and Figure 22. The plots show a small bias between the two systems. In most cases, the bias is less than one degree, with the abduction angle at trot as a notable exception at about two degrees. The limits of agreement show similar performance for protraction and retraction angles at walk and trot, with a deviation between the systems ranging up to three degrees in most cases. The regression lines in the plot show no clear trend and no obvious indication of heteroscedasticity. In contrast, the adduction and abduction angles show significantly less agreement: while the bias is still relatively small for all coronal angles, the limits of agreement show a deviation of up to nine degrees between the two systems. We speculate once again that the relatively poor coronal agreement is due to the poor depth perception of the OMC system.

For the parameters tested, the regression lines in the Bland–Altman plots seem to indicate that the bias is relatively constant throughout the measured ranges; therefore, no heteroscedasticity was visible. The small slope in some of the plots is caused by the fact that there is a significant difference between fore- and hindlimbs, which causes clustering in the data, particularly for the retraction at walk and trot (see Figure 20).

**Figure 23:**
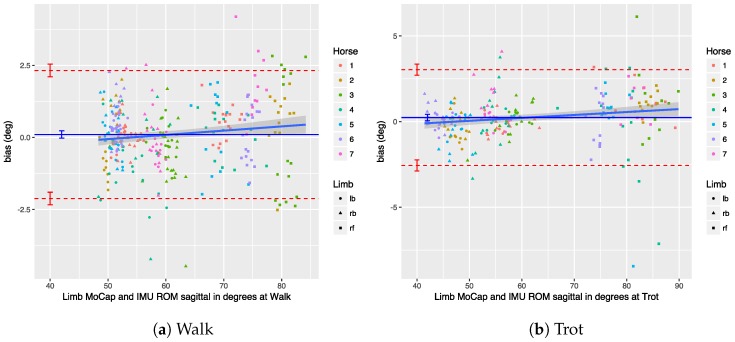
Bland–Altman analysis for sagittal range of motion (ROM) at walk and trot.

**Figure 24:**
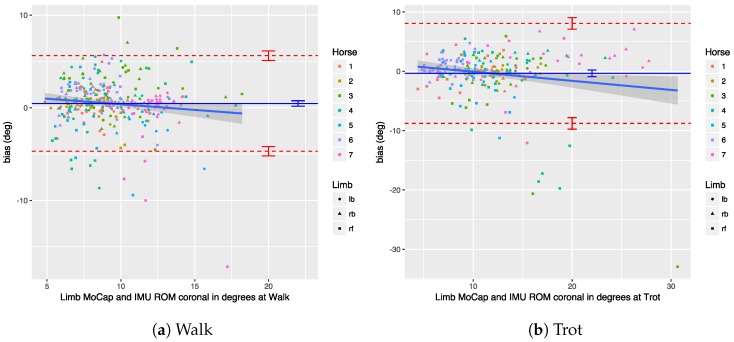
Bland–Altman analysis for coronal ROM at walk and trot.

We performed a Bland–Altman analysis of the angular range of motion (ROM) for the sagittal and coronal planes (see Section 3.2.2), respectively in Figure 23 and Figure 24. The bias is mostly insignificant, with the confidence interval (nearly) containing zero. The agreement is within around 2.5 degrees for the sagittal plane. Again, the coronal plane performs worse at five degrees for walk and about eight degrees for trot.

A summary of the agreement statistics for the sagittal and coronal angular limb parameters is presented in Table 3, Table 4, Table 5 and Table 6 for walk and trot. The agreement is presented in the tables both per stride and per trial.

Results indicate that in most cases the OMC and IMU systems agreed within 2–3 degrees for protraction, retraction, and sagittal ROM measurements. In horses with gait abnormalities, angular changes greater than three degrees have been described [90], so—considering the OMC as a validity reference—the IMU system can theoretically measure biologically significant differences. The picture is less clear for the adduction angles, abduction angles, and coronal ROM, since the agreement interval is at worst ±9 degrees for those. We theorize that the poor agreement is due to problems in the OMC system, mainly caused by depth perception of the cameras [89], although there is no way to prove that with the present data set. As discussed, for the forelimbs, the placement of the markers may also be a factor.

The reason why the per trial results are presented here is to compare these with earlier work by Roepstorff et al. [69]. As described in Section 2, the per trial results may prove to be too optimistic about the agreement. The tables show this effect quite clearly. The per trial results are always almost twice better than the per stride agreement, demonstrating this problem. From plots in the paper by Roepstorff et al., we estimate the limits of agreement for their experiment to be within about two degrees. They only present sagittal ROM measurements, for which our system consistently performed better, both in terms of bias and limits of agreement.

The ICC results show high reproducibility of the measurements between the two systems (close to 1) for almost all experiments, with the coronal angles for the forelimbs as a notable exception. This is in line with the Bland–Altman analysis, where we also observed narrow limits of agreement, indicating a good agreement between the two instruments. As before, we speculate that the lower ICC observed for the angles in the coronal plane are related to the poor depth perception of the OMC due to the camera set-up in our venue.

### 7.4. Upper-Body Symmetry

Table 7 presents difference statistics for the upper-body vertical displacement signals. The root mean square error (RMSE), mean residual, and standard deviation (S.D.) of residuals at both withers and sacrum indicate that the vertical displacement signal measured by the two systems is highly comparable. The mean of residuals is zero, indicating that there is no bias between the two systems on a signal level. As seen earlier in our ICC and Bland–Altman analysis, the coronal results for RMSE and standard deviation of residuals demonstrate a poorer performance when compared to the sagittal angles. As previously discussed, this could be attributed to the poor depth perception of the cameras. Nevertheless, these results demonstrate a good reproducibility between the two systems, not only taking into account the moments used to calculate the gait parameters (i.e., peaks and valleys), but also along the entire signal.

Earlier work by Warner et al. [32] evaluated the signal differences between a generic IMU-based system and an OMC system. The authors reported a mean difference in the vertical displacement signal of 0.0 mm (S.D. 2.3 mm) at the withers and 0.0 mm (S.D. 4.1 mm) at the sacrum. These results are in line with the corresponding results of our study in Table 7, although we achieved a better precision, especially at the sacrum. This difference may be attributed to the lower sampling frequency used by Warner et al. (100 Hz) and possibly due to differences in the applied filters.

**Figure A3:**
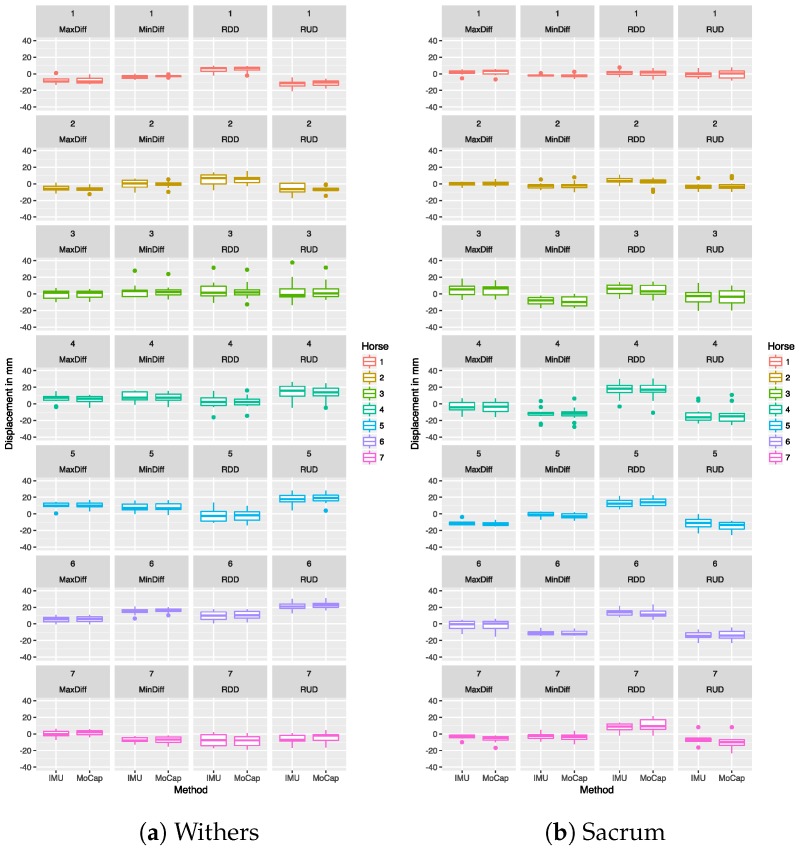
Box plot for the upper-body vertical displacement parameters of IMU and OMC.

Figure A3 in Appendix A shows box plots for the statistics of the differences between the IMU and OMC systems for the upper-body symmetry parameters (see Section 3.2.3) at the withers and sacrum, respectively. The statistics are presented for the individual horses. In most cases, the statistics are similar between the two systems, apart from a few outliers.

**Figure 25:**
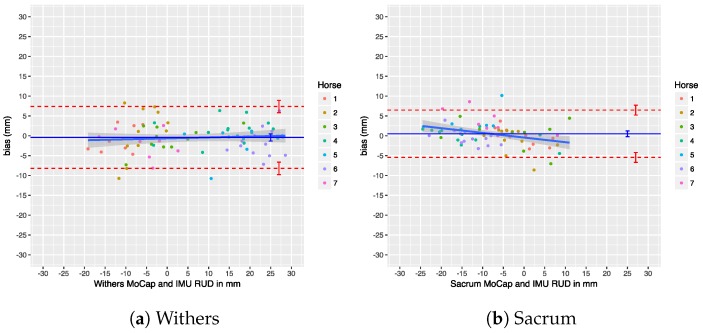
Bland–Altman analysis for $\mathrm{Range}\_{\mathrm{diff}}_{\mathrm{up}}$ parameter.

**Figure 26:**
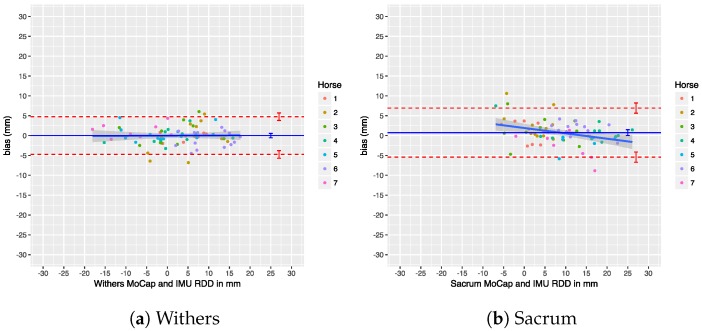
Bland–Altman analysis for $\mathrm{Range}\_{\mathrm{diff}}_{\mathrm{down}}$ parameter.

**Figure 27:**
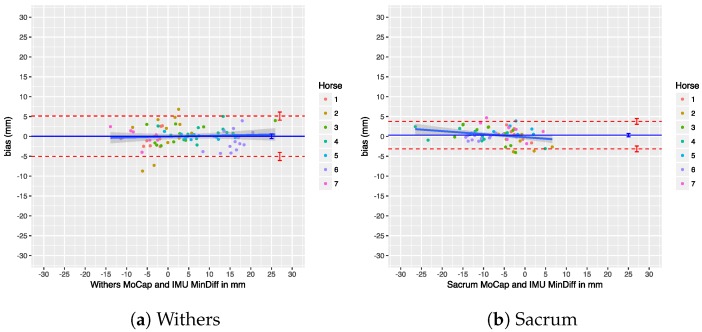
Bland–Altman analysis for Min_diff parameter.

**Figure 28:**
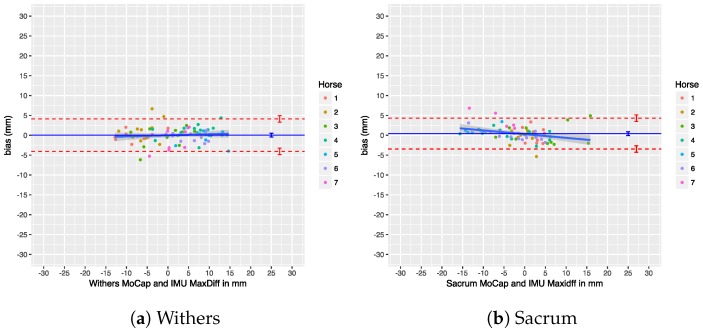
Bland–Altman analysis for Max_diff parameter.

We performed a Bland–Altman analysis of the $\mathrm{Range}\_{\mathrm{diff}}_{\mathrm{up}}/\mathrm{down}$ parameters for the withers and sacrum in Figure 25 and Figure 26. The agreement of the Min_diff and Max_diff parameters at those locations is shown in another set of Bland–Altman plots in Figure 27 and Figure 28. Overall, there is no significant bias in the agreement between the two systems: the zero line lies within the confidence interval of each bias line. The limits of agreement show that in most cases and for most parameters the systems agree within about 5 mm. The regression lines in the Bland–Altman plots show no clear trends. A summary of the agreement statistics is presented in Table 8 and Table 9 for the the withers and sacrum, respectively.

It would be useful to compare our agreement results with existing systems: the Lameness Locator [27,37,72] and EquiGait [34,74] systems both yield upper-body symmetry parameters. However, to the best of our knowledge, there is no earlier work presenting an ICC or Bland–Altman analysis that compares either of these systems to some form of (gold-standard) reference system. Therefore, our results are difficult to compare with systems already on the market. One previous study by Warner et al. [32] evaluated the agreement between a generic IMU-based system and an OMC system. However, since the Bland–Altman agreement analysis is performed on signals rather than parameters, we have no common ground to compare our results with theirs.

Recently, an agreement analysis between the Lameness Locator and the EquiGait systems was performed on a trial level (trial mean) [67]. The authors found not only wide limits of agreement for the straight line trot (Min_diff poll ±6.4 mm and Min_diff sacrum 4.4 mm), but also a systematic bias between the systems. Our results for agreement between OMC and our IMU-based system have a better agreement. It is important to mention that, in the work of Pfau et al. [67], some differences between the two systems might explain their findings: mainly the different filters used by the two systems (EquiGait uses an infinite impulse response (IIR) filter and the Lameness Locator uses a signal decomposition based on a curve fitting method [82]). Additionally, the EquiGait system determines displacement in a global coordinate frame, while the Lameness Locator uses a local coordinate frame, which is particularly prone to deviations when the sensor is not aligned with the horse’s vertical axis (which happens during locomotion).

Previous research has used thresholds to declare horses as lame or sound, based on the level of measured asymmetry, although this has recently been criticized [1]. Nevertheless, if we take these thresholds into consideration, our limits of agreement for withers and sacrum on a trial level (see Table 8 and Table 9) are still within the proposed threshold of less than 6 mm for Min_diff poll and less than 3 mm for Min_diff sacrum (although we cannot actually compare poll results directly, as described in Section 5.3). This indicates that, on a trial level, our system can be used to quantify motion asymmetry within the required resolution.

Additionally, our system shows a bias of less than 1 mm for all calculated symmetry parameters, which makes comparison of calculated symmetry parameters between the two systems possible. This is especially important for reproducibility and when comparing research output using different systems to measure motion symmetry, which increases the reproducibility of scientific work. Furthermore, if a patient is measured by different veterinarians, using these two different systems, the results can be readily compared without the need of repeating measurements.

## 8. Future Work

Based on experience gained from these experiments, the experimental setup should be adjusted in a few points for future experiments. Firstly, the handler needs to be farther away from the horse to prevent OMC marker occlusion on the forelimbs. Additionally, the placement of the markers on the forelimbs is too linear—the markers need to be spread more. The OMC system company provides special rigid marker clusters now, which should be used instead of placing markers individually. Furthermore, the IMU and/or OMC markers at the poll should be moved such that a reliable comparison between the systems is possible for that location. Finally, it is better to also have cameras closer to the ground to capture limb motion reliably. However, expanding the OMC system is costly.

In future experiments, we aim to calculate more horse locomotion parameters. For the limbs, we currently only measure angles, but it is also useful to determine limb displacement parameters such as stride length, average stride speed, and the maximum hoof elevation [91]. Conversely, for the upper body, we currently rely solely on parameters based on vertical displacement. However, for example, the pelvis roll angle, which is the coronal attitude of the pelvis relative to the horizontal, has merit as well [92].

The scope of future experiments will be extended. In the present study, we performed an agreement analysis involving only horses that have no known locomotion issues. To perform actual lameness detection, we will perform a proof-of-concept study with induced lameness. Furthermore, it is important to evaluate our system and algorithms with gaits other than walk and trot, and trials with horses moving in a nonlinear path; e.g., circular (lungeing) [9,33,93].

## 9. Conclusions

The agreement between the OMC and EquiMoves systems was overall good, except for the adduction and abduction. We attribute this to the setup of the OMC system: the cameras have difficulty with depth perception at this distance, and the marker placement at the forelimbs was not optimal. This may in fact highlight the strength of IMU-based systems, especially for measuring gait variables that are not easily tracked by OMC systems.

The results suggest that the EquiMoves system can be a good aid for veterinarians during gait analysis (e.g., for lameness exam [10] or sports performance analysis [94]). It can measure several important parameters within a level of accuracy that is capable of detecting gait changes due to lameness or performance degradation.

## Figures and Tables

**Table 1:** Properties of the horses involved in the experiments.

Horse	Weight (kg)	Height (m)	Age (years)
**1**	534	1.69	12
**2**	512	1.62	11
**3**	593	1.61	10
**4**	578	1.66	10
**5**	514	1.65	16
**6**	593	1.66	21
**7**	587	1.66	14
**Mean**	559	1.65	13

**Table 2:** Summary of difference statistics for the limb angle signals (values in degrees). RMSE: root-mean-square error; S.D: standard deviation.

	Walk				Trot			
	Sagittal		Coronal		Sagittal		Coronal	
	Forelimb	Hindlimb	Forelimb	Hindlimb	Forelimb	Hindlimb	Forelimb	Hindlimb
RMSE	0.67	0.67	1.27	1.01	1.05	0.95	1.79	1.21
Mean Residual	0.02	0.02	−0.06	−0.03	−0.03	−0.02	−0.03	0.04
S.D. of Residuals	0.25	0.24	0.43	0.34	0.55	0.50	0.95	0.66

**Table 3:** Summary of measurement agreement statistics for the sagittal limb angles at walk (values in degrees). ICC: intra-class correlation; LOA: limit of agreement; ROM: range of motion.

	Retraction		Protraction		Sagittal ROM	
	Per Stride	Trial Mean	Per Stride	Trial Mean	Per Stride	Trial Mean
Upper LOA	1.70	−0.75	1.61	0.84	2.32	1.29
Lower LOA	−2.29	−1.30	−1.98	−1.15	−2.12	−1.06
Bias	−0.29	−0.27	−0.19	−0.16	0.10	0.12
ICC R. Forelimb	0.99		0.99		0.99	
ICC R. Hindlimb	0.99		0.97		0.99	
ICC L. Hindlimb	0.99		0.98		0.99	
ICC Final	0.99		0.98		0.99	

**Table 4:** Summary of measurement agreement statistics for the coronal limb angles at walk (values in degrees).

	Adduction		Abduction		Coronal ROM	
	Per Stride	Trial Mean	Per Stride	Trial Mean	Per Stride	Trial Mean
Upper LOA	4.22	2.51	5.61	3.14	5.62	3.41
Lower LOA	−2.93	−1.36	−4.69	−2.73	−4.69	−2.72
Bias	0.64	0.58	0.46	0.34	0.46	0.34
ICC R. Forelimb	0.91		0.82		0.81	
ICC R. Hindlimb	0.87		0.98		0.98	
ICC L. Hindlimb	0.98		0.96		0.97	
ICC Final	0.92		0.92		0.92	

**Table 5:** Summary of measurement agreement statistics for the sagittal limb angles at trot (values in degrees).

	Retraction		Protraction		Sagittal ROM	
	Per Stride	Trial Mean	Per Stride	Trial Mean	Per Stride	Trial Mean
Upper LOA	1.79	1.07	2.27	1.17	3.03	1.52
Lower LOA	−2.81	−2.04	−2.81	−2.09	−2.56	−0.97
Bias	−0.50	−0.48	−0.27	−0.21	0.25	0.27
ICC R. Forelimb	0.96		0.97		0.97	
ICC R. Hindlimb	0.98		0.96		0.99	
ICC L. Hindlimb	0.97		0.98		0.99	
ICC Final	0.97		0.97		0.98	

**Table 6:** Summary of measurement agreement statistics for the coronal limb angles at trot (values in degrees).

	Adduction		Abduction		Coronal ROM	
	Per Stride	Trial Mean	Per Stride	Trial Mean	Per Stride	Trial Mean
Upper LOA	5.39	2.32	8.07	4.05	8.07	4.06
Lower LOA	−5.14	−2.09	−8.78	−4.62	−8.79	−4.62
Bias	0.12	0.12	−0.36	−0.28	−0.36	−0.28
ICC R. Forelimb	0.79		0.86		0.86	
ICC R. Hindlimb	0.92		0.99		0.99	
ICC L. Hindlimb	0.98		0.98		0.98	
ICC Final	0.90		0.94		0.94	

**Table 7:** Summary of difference statistics for the upper-body vertical displacement signals (values in mm).

	Withers	Sacrum
RMSE	2.43	1.85
Mean Residual	0.01	−0.02
S.D. of Residuals	2.43	1.85

**Table 8:** Summary of measurement agreement statistics for the withers (values in mm).

	Min_diff		Max_diff		$\mathrm{Range}\_{\mathrm{diff}}_{\mathrm{up}}$		$\mathrm{Range}\_{\mathrm{diff}}_{\mathrm{down}}$	
	Per Stride	Trial Mean	Per Stride	Trial Mean	Per Stride	Trial Mean	Per Stride	Trial Mean
Upper LOA	5.00	1.40	4.10	1.70	8.20	2.01	4.70	1.63
Lower LOA	−5.10	−1.30	−4.10	−1.76	−7.40	−3.00	−4.80	−1.45
Bias	0.04	0.05	0.02	−0.03	0.43	−0.47	0.01	0.09
ICC	0.98		0.98		0.99		0.97	

**Table 9:** Summary of measurement agreement statistics for the sacrum (values in mm).

	Min_diff		Max_diff		$\mathrm{Range}\_{\mathrm{diff}}_{\mathrm{up}}$		$\mathrm{Range}\_{\mathrm{diff}}_{\mathrm{down}}$	
	Per Stride	Trial Mean	Per Stride	Trial Mean	Per Stride	Trial Mean	Per Stride	Trial Mean
Upper LOA	3.20	2.09	3.50	2.61	5.40	4.59	3.90	4.05
Lower LOA	−3.80	−1.16	−4.30	−1.47	−6.50	−2.90	−4.50	−3.18
Bias	0.31	0.46	0.41	0.57	0.51	0.85	0.30	0.43
ICC	0.94		0.95		0.91		0.94	

## Appendix A

This Appendix contains box plots for the sagittal and coronal limb angles measured by IMU and OMC in Figure A1 and Figure A2, respectively. Additionally, box plots for the the upper body vertical displacement parameters of IMU and OMC are presented in Figure A3.
